# Menaquinone Content of Cheese

**DOI:** 10.3390/nu10040446

**Published:** 2018-04-04

**Authors:** Cees Vermeer, Joyce Raes, Cynthia van ’t Hoofd, Marjo H. J. Knapen, Sofia Xanthoulea

**Affiliations:** R&D Group VitaK, Maastricht University, Oxfordlaan 55, 6229 EV Maastricht, The Netherlands; cees.vermeer@outlook.com (C.V.); j.raes@vitak.com (J.R.); c.vanthoofd@vitak.com (C.H.); sofia.xanthoulea@maastrichtuniversity.nl (S.X.)

**Keywords:** vitamin K, menaquinone, cheese, diet, cardiovascular disease

## Abstract

Vitamin K_2_ (menaquinone) concentrations were measured in a wide range of cheeses and the effects of fat content, ripening and origin of the cheeses were investigated. Moreover, the menaquinone content of cheese was compared with that of other foods known to contain vitamin K_2_. It was found that cheese and curd are the most important sources of long-chain menaquinones in the Western diet and, in general, hard cheeses are richer in menaquinones than soft cheeses. However, the actual menaquinone content varies substantially and is dependent on the type of cheese, the time of ripening, the fat content and the geographic area where the cheeses are produced. Given the fact that poor vitamin K status has been mentioned as a risk factor for cardiovascular disease and mortality, while there is no clear evidence for adverse cardiovascular effects of dairy fats, cheese should be considered as a recommendable component in a heart-healthy diet.

## 1. Introduction

Vitamin K is a micronutrient that serves an essential role as a cofactor for the γ-carboxylation of glutamic acid residues in a number of proteins. This modification is essential for the proper function of these proteins in blood coagulation and in the regulation of bone and vascular calcium homeostasis [1,2,3]. 

Vitamin K occurs in different forms that all share a common ring structure called 2-methyl-naphthoquinone (also known as menadione), but differ in the composition of the poly-isoprenoid side chain attached at the 3-position. Phylloquinone (also known as vitamin K_1_) is synthesized by green plants, and has a side chain of 4 isoprenoid residues of which the first one is unsaturated. The most abundant dietary sources are green, leafy vegetables such as spinach, kale, broccoli and sprouts [4,5]. Menaquinones (collectively known as vitamin K_2_) differ from each other in the length of their side chains at the 3-position (MK-n, in which the suffix n stands for the number of isoprenoid residues in the side chain). Their distribution in human nutrition is more restricted than phylloquinone, the most abundant source in the Western diet being fermented dairy products such as cheese and curds [6]. 

The relative importance of menaquinones in food is larger than generally assumed. The underlying reason is that intestinal absorption of phylloquinone from vegetables is 5–10%, whereas uptake of menaquinones from dairy products is almost complete [7]. Whereas dietary phylloquinone forms about 90% of the total vitamin K intake, the relative contribution of menaquinones to human vitamin K status is at least equal to that of phylloquinone. 

In the past decade, increasing attention has been paid to the health benefits of the K_2_ vitamins, notably the long-chain menaquinones MK-7, MK-8 and MK-9 [8,9,10,11,12,13,14]. Both phylloquinone and menaquinones are absorbed (together with dietary fat) from the small intestine and transported in the circulation by lipoproteins [15]. The majority of circulating phylloquinone is associated with the triacylglycerol-rich lipoproteins (TRL) which are rapidly cleared by the liver [2]. The long-chain menaquinones, however, can circulate for extended times because of their association with low-density lipoproteins (LDL). Consistent with the different pharmacokinetics of TRL and LDL, the half-life times of phylloquinone and MK-7 are 1.5 h and 3 days, respectively [2,16]. The difference in transport may have implications for their bioavailability and tissue distribution, and most likely explains the higher efficacy of MK-7 compared to phylloquinone [16]. Therefore, long-chain MKs are thought to be the most adequate form of vitamin K to supply LDL receptor-containing extra-hepatic tissues such as the arterial vessel wall and bone. This may also explain the inverse associations found between dietary menaquinones (but not phylloquinone) and the risk of cardiovascular disease and cancer [8,9,10].

With the growing interest in health benefits of the long-chain menaquinones [17], it is important to have elaborate and high quality up-to-date data on their concentration in human nutrition. Since cheese was shown to be the richest source of menaquinones in the human diet [6], we have focused in this paper on the menaquinone content of different forms of cheese. We have compared the vitamin K content of the most common Dutch cheeses as well as the effect of ripening and fat content; we have also examined cheeses from various geographic areas in Europe, and their relative vitamin K content as compared with other food products. The food composition tables thus obtained may be used in calculating vitamin K intake from food frequency questionnaires in population-based studies.

## 2. Materials and Methods 

### 2.1. Origin of Foods

Most products mentioned in this paper were purchased in 2013 from Dutch supermarkets (industrial Dutch cheeses), organic food stores (raw milk cheeses) and specialized food shops (foreign cheeses) for analysis of their vitamin K profile, i.e., the amount and relative proportion of the different K vitamins. A broad panel of popular Dutch cheeses was selected, varying in fat content and duration of ripening. These cheeses were compared with cheeses from different European countries including Greece, Switzerland, UK, Norway, France and Italy. We also purchased various raw-milk farm cheeses to compare their values with those of the industrially prepared supermarket cheeses. Besides cheese, we also analyzed a number of other menaquinone-containing products including meat and fish (all bought in local supermarkets). All food products were stored at 4 °C and processed within 1 week or stored at −20 °C and processed within 3 weeks. The fat contents given throughout this paper refer to the content as given on the food labels.

### 2.2. Vitamin K Analysis

Vitamin K was quantified according to previously described procedures with some small adaptations for fat cheese [6]. Briefly, samples were supplemented with the appropriate amount of internal standard vitamin K_1_(25) (GL Synthesis, Worcester, MA, USA) and extracted with chloroform (Biosolve BV, Valkenswaard, The Netherlands)-methanol (Biosolve BV, Valkenswaard, The Netherlands) (1:1, *v*/*v*). If required, a lipase digestion at 37 °C preceded the extraction step. Extracts were evaporated to dryness under a constant stream of nitrogen, dissolved in hexane and pre-purified by solid phase extraction on silica columns. The eluate was reconstituted in 80 μL isopropanol and analyzed for vitamin K by HPLC (High Performance Liquid Chromatography) using a C-18 reversed phase column (Thermo Scientific, Waltham, MA, USA) and fluorometric detection after post-column reduction using a zinc column [6]. Phylloquinone and menaquinones were recorded in the same run. All data given are means of duplicate samples that were analyzed separately. The variation coefficient was <15% and the detection limit was 10 pg per injection for each vitamer. Authentic menaquinones MK-4 through MK-10 were a kind gift from Roche (Basel, Switzerland) and served as reference compounds.

## 3. Results

A number of Dutch cheeses of different fat content and ripening periods were analyzed for their phylloquinone (vitamin K_1_) and menaquinones (vitamin K_2_) content (Table 1). In all cheeses it was found that the very young ones had a slightly lower menaquinone content than the older ones and after 13 weeks of ripening a certain plateau level was reached. This difference is mainly due to the increased levels of long-chain menaquinones in more ripened cheeses, which originate from bacterial growth during fermentation. It was found that in the most popular full fat cheese (Gouda 13 weeks, 50% dry weight fat) the menaquinone content was around 650 ng/g while in the very young cheese (Gouda 4 weeks) it was substantially lower (473 ng/g). No significant difference was found between freshly cut cheese at a stretch and vacuum-packed products. Demi-fat cheese (Milner 4 weeks, 13 weeks or 26 weeks, 30% dry weight fat) had a lower menaquinone content independently of the ripening duration (around 450 ng/g), and low-fat cheese (Slankie 4 weeks, 13 weeks or 26 weeks, 20% dry weight fat) contained not more than approximately 380 ng/g. Edam (40% dry weight fat) and Maasdam (5 weeks, 45% dry weight fat) cheeses were relatively rich in menaquinones with 647 ng/g and 490 ng/g respectively, whereas curd cheese contained between 94 and 140 ng/g, independent of the fat content. Raw milk cheese (not industrially prepared but originating from local farms) was rich in menaquinones (between approximately 600 and 790 ng/g). 

When comparing cheeses from different countries, remarkable differences were observed (Table 2). Notably, cheeses produced in Mediterranean countries (feta, mozzarella, gorgonzola, pecorino) were low in menaquinone content with Parmesan cheese containing almost negligible amounts (3 ng/g). French cheeses, Brie and Boursin, had low vitamin K_2_ content (125 ng/g and 111 ng/g, respectively), whereas it was considerably higher Camembert and Roquefort (681 ng/g and 381 ng/g, respectively). The highest content of menaquinones in French cheeses was measured in Münster cheese (originating from the Vosges and made from raw, unpasteurized milk) with 801 ng/g total vitamin K_2_. 

Both British cheeses measured (Cheddar and Stilton) contained average to high amounts of menaquinones (235 ng/g and 494 ng/g, respectively) as did Swiss cheeses, Emmenthal (433 ng/g) and Raclette (323 ng/g), while Gruyère contained low amounts (65.3 ng/g). Remarkably, the Swiss Emmental cheese almost exclusively contained MK-10, which is produced by the probiotic *Propionibacterium freudenreichii* that is also thought to be responsible for the large holes and the typical taste of this cheese. However, it was also reported that these large holes could be due to the presence of hay particles during cheese maturation. Finally, two Norwegian cheeses measured, Gamalost and Norvegia, contained relatively high amounts of menaquinones with 542 ng/g and 415 ng/g, respectively.

To be able to evaluate the importance of cheese as a source of vitamin K_2_, we measured the menaquinone content in a number of other foods known to contain menaquinones like meat, fish and vegetables (Table 3). It is clear that cheese is the richest source of vitamin K_2_ in the Western diet and almost the only source of the long-chain menaquinones. Meat contained almost exclusively MK-4, probably originating from the covalent bonding of geranylgeraniol to the 3-position of menadione (a synthetic product often added to animal food). Only liver contained some long chain menaquinones, but this is not consumed on a large scale. The rather high MK-4 content of eel is at variance with previous observations [6] and may be explained by the fact that nowadays eel is cultured and receives menadione-rich food, whereas 18 years ago eel caught in the wild was analyzed. Among vegetables, only the two fermented ones, natto and sauerkraut, contained vitamin K_2_, with the total menaquinone content of the latter being 200 fold lower than that of natto, known to be an important source of menaquinones (MK7) in Japan.

## 4. Discussion

In this paper we report the menaquinone content of various cheeses and foods. The typical Dutch hard cheeses were rich in menaquinones, whereas some typical Mediterranean cheeses contained very small amounts. Because of these differences, menaquinone intake may depend on personal preferences and may also differ significantly from one country to the other. Therefore, it is important to have national databases for menaquinone content of food. This is important to realize when using food composition tables in population-based studies on vitamin K intake.

Several techniques for quantification of K vitamins have been reported in the literature. In common, they have organic solvent extraction of the sample, followed by pre-purification and separation by reversed-phase HPLC or UPLC (Ultra Performance Liquid Chromatography). Three detection methods are currently used: post-column reduction with either fluorescent [18] or electrochemical detection [19], and mass spectrometry [20,21]. Whereas the first method is presently the most widely used, detection techniques based on mass spectrometry are generally more sensitive than other detection methods. For the analyses presented here, we have used fluorescent detection, which is sufficiently sensitive to demonstrate the different menaquinone contents in various foods. Various internal standards have been reported, including MK-6, but presently vitamin K_1_(25) is the most common and reliable one; it is highly similar to phylloquinone, but its side chain contains one extra isoprenoid residue. Authentic phylloquinone and menaquinones should be used as reference compounds for calibration purposes; in this respect, the availability of authentic menaquinones is critical because not all are commercially available. For this study, we have chosen to use detection based on fluorescence after post-column reduction and authentic menaquinones MK-4 through MK-10 served as reference compounds. 

The major forms of vitamin K in dairy products were MK-9 followed by MK-4 and MK-8. MK-8 and MK-9 are both produced by the microbes in the starter ferment and are highly inter-correlated (*r* = 0.9); for most dairy products, the level of MK-9 was about four times higher than that of MK-8, suggesting that both forms of vitamin K are produced in a constant ratio. MK-10 was found only in Emmental cheese and probably originates from the *Propionibacterium freudenreichii* used in the starter ferment. Remarkably, the Dutch Maasdam cheese (which is also prepared with a propionibacteria-containing ferment) did not contain MK-10. This suggests that MK-10 production by different sub-strains of propionibacteria may differ considerably.

Remarkably, the menaquinone content of typical Dutch hard cheeses (Gouda, Edam) was relatively high compared to most soft cheeses as produced in Mediterranean countries. This is in contrast to a recent study from the USA [22], in which soft cheeses were found to contain on average about twice as much menaquinones as hard cheeses. This demonstrates the necessity of having national databases for menaquinone content of food.

Except for natto (see below), cheese was the nutrient with the highest menaquinone concentrations. However, even the highest amount of menaquinone found in this study (801 ng/g, Münster cheese), was moderate compared to the levels of phylloquinone found in green leafy vegetables (up to 8 µg/g) [6]. Considering the fact that the intestinal absorption of phylloquinone from vegetables like kale, spinach and broccoli is between 5 and 10% of that from food supplements, whereas menaquinone uptake from dairy is much better and comparable to that from oil-based products or supplements [7,23,24], it may be calculated that menaquinone is at least equally important for vitamin K status as phylloquinone. Another important point is that due to different pharmacokinetics, menaquinones are more important for extrahepatic tissues than phylloquinone [16].

The menaquinone content of other foods (meat, fish) was low, consisted mainly of MK-4 and cannot be expected to significantly contribute to vitamin K status, also because of the short half-life time of MK-4 [7,16]. The exception is natto, a traditional Japanese food consisting of fermented soybeans, which is extremely rich in MK-7; however, because of its strong taste and smell, natto is not appreciated outside Japan. In a recent paper from the USA [25], it was shown that pork contains considerable amounts of MK-4, MK-10 and MK-11. Whereas MK-11 has not been measured in our study, these data are not consistent with our (limited) data on meat, notably pork. The difference may be related to different animal food used on the farms. Hence in Western Europe, cheese is the main source of the long-chain menaquinones, which were reported to contribute to cardiovascular disease prevention [8,9,26]. Also, poor vascular vitamin K status, as concluded from high levels of circulating inactive MGP (Matrix Gla Protein), was found to be a risk factor for cardiovascular disease and mortality [11,12,13,27,28,29,30,31]. In an open-label single-arm study among 26 patients with established high risk for CAC (Coronary Artery Calcification), however, CAC progressed by 14% annually despite high dose MK-4 supplementation. The arterial stiffness in this study had not changed overall, but this was only due to a decreased stiffness in patients with baseline vitamin K insufficiency [32]. 

Although cheese was found to be the most important source of dietary long-chain menaquinones, and long-chain menaquinones have been reported to be associated with cardiovascular risk reduction, the question is warranted whether cheese (notably hard cheese) should be recommended as part of a healthy lifestyle. Especially in the light of the high content of saturated fat, which for a long time has been regarded as unhealthy. Recently, however, the unhealthy aspects of dairy fat have been questioned and reappraisals of the impact of dairy foods and milk fat on cardiovascular disease risk concluded there is no clear evidence that cheese consumption is consistently associated with a higher risk of cardiovascular disease [33,34]. De Goede et al. performed a meta-analysis including 18 studies with 8 to 26 years of follow-up that included 762,414 individuals and 29,943 stroke events [34]. The authors concluded that cheese consumption is inversely associated with stroke risk, with 3% lower risk of stroke per 40 g/day of cheese consumption. Remarkably, both high and low fat dairy were associated with lower stroke risk. In another meta-analysis, De Souza et al. concluded that saturated fats are not associated with all-cause mortality, CVD (Cardiovascular Disease), CHD (Congenital Heart Disease), ischemic stroke, or type-2 diabetes, but that the evidence is heterogeneous with methodological limitations [35]. In another study, Chowdhury and colleagues compared 32 observational studies on fatty acids from dietary intake, 17 observational studies on fatty acid biomarkers and 27 randomized, controlled trials of fatty acid supplementation [36]. In the observational studies, relative risks for coronary disease were insignificant for saturated, for long-chain ω-3 polyunsaturated, and for ω-6 polyunsaturated fatty acids. In the randomized controlled trials, relative risks for coronary disease were 0.97 (CI (Confidence Interval), 0.69 to 1.36) for α-linolenic, 0.94 (CI, 0.86 to 1.03) for long-chain ω-3 polyunsaturated, and 0.89 (CI, 0.71 to 1.12) for ω-6 polyunsaturated fatty acid supplementations. From these and other data [37], it was concluded that current evidence does not support cardiovascular guidelines that encourage high consumption of polyunsaturated fatty acids and low consumption of total saturated fats.

Limitations of our study are: 

1. Our tables are not exhaustive; only a few examples of the most common cheeses have been analyzed.

2. The foreign cheeses were not bought in the country of origin but in the local stores in Maastricht.

3. The effect of ripening was investigated only in one type of cheese and may be different in other cheeses. It should be pointed out that ripening is a well-controlled process taking place at the site of production. Whether the menaquinone content may depend on shelf-life time should be considered in future investigations.

Despite these limitations, we believe that this study gives a representative picture of the vitamin K content of cheeses and curds.

## 5. Conclusions

In conclusion, we have found that cheese and curd are the most important sources of long-chain menaquinones in the Western diet but that the actual menaquinone content varies substantially and is dependent on the type of cheese, the time of ripening, the fat content and the geographic area where this is produced. In general, hard cheeses contain more menaquinones than soft cheeses. Given the fact that several authors have reported poor vitamin K status to be a risk factor for cardiovascular diseases while the most recent meta-analyses suggest no clear evidence for adverse cardiovascular effects of dairy fats, cheese should be considered as a recommendable component in a heart-healthy diet.

## Figures and Tables

**Table 1:** Vitamin K content of Dutch cheeses. The most popular cheese is Gouda after 13 weeks of ripening. Milner is a comparable cheese with lower fat content and Slankie is a very low fat product line. Raw milk cheese was produced at local farms; the curds were from brands Albert Heijn (AH) and Friesland Campina (FC). Fat content of cheeses is given as % dry weight; fat content of curds is given as grams per 100 mL.

Type of Cheese	Fat (%)	Vitamin K_1_ (ng/g)	MK-4 (ng/g)	MK-5 (ng/g)	MK-6 (ng/g)	MK-7 (ng/g)	MK-8 (ng/g)	MK-9 (ng/g)	MK-10 (ng/g)	Total Vitamin K_2_ (ng/g)
Gouda 4 weeks	50	37.6	145	4.3	4.8	14.8	72.0	232	0.0	473
Gouda 13 weeks	50	39.6	148	3.8	4.3	15.9	87.6	396	0.0	656
Gouda vacuum 13 weeks	50	34.4	115	3.6	4.7	17.3	94.2	424	0.0	659
Gouda 26 weeks	50	39.4	208	4.2	4.8	16.2	92.8	403	0.0	729
Gouda vacuum 26 weeks	50	37.7	154	4.3	4.9	15.7	96.8	368	0.0	644
Milner 4 weeks	30	22.0	77.6	3.5	3.0	9.6	58.0	284	0.0	436
Milner 13 weeks	30	23.1	84.1	4.1	3.4	10.7	65.2	306	0.0	474
Milner 26 weeks	30	22.8	102	4.2	3.7	10.6	62.8	268	0.0	451
Slankie 4 weeks	20	17.2	80.9	3.6	4.0	9.9	42.4	192	0.0	333
Slankie 13 weeks	20	19.0	64.2	4.3	4.5	8.9	37.8	150	0.0	270
Slankie 26 weeks	20	19.8	78.9	4.9	5.4	9.8	54.5	233	0.0	387
Edam	40	37.6	113	0.0	0.0	0.0	74.6	459	0.0	647
Maasdam 5 weeks	45	35.3	115	4.5	4.7	14.5	84.8	266	0.0	490
Whole curd FC	8.8	9.4	22.6	4.8	2.8	5.5	13.9	44.3	0.0	94
Whole curd AH	8	12.9	25.7	2.3	1.4	4.0	20.5	81.4	0.0	135
Demi-skimmed curd AH	3	4.9	15.5	1.8	1.5	5.3	19.0	97.3	0.0	140
Jersey (from raw milk)	50	42.8	60.3	16.2	13.9	38.4	154	506	0.0	789
Loverendale (from raw milk)	50	46.8	166	16.5	6.3	13.2	87.4	315	0.0	604

Each value is the average value of duplicate measurements with a variation coefficient < 15%.

**Table 2:** Vitamin K content of various European cheeses.

Type of Cheese	Vitamin K_1_ (ng/g)	MK-4 (ng/g)	MK-5 (ng/g)	MK-6 (ng/g)	MK-7 (ng/g)	MK-8 (ng/g)	MK-9 (ng/g)	MK-10 (ng/g)	Total Vitamin K_2_ (ng/g)
**French Cheeses**									
Brie	49.2	125	0.0	0.0	0.0	0.0	0.0	0.0	125
Boursin	45.5	89.3	0.0	1.1	3.3	8.2	9.1	0.0	111
Camembert	25.0	79.5	13.4	10.1	32.4	151	395	0.0	681
Roquefort	65.6	131	6.4	4.8	11.6	50.9	176	0.0	381
Münster	20.6	102	4.5	4.6	83.7	412	194	0.0	801
**British Cheeses**									
Cheddar	21.6	51.2	0	3.8	18.8	36.4	125	0.0	235
Stilton	36.2	100	9.4	6.0	14.0	66.3	298	0.0	494
**Greek Cheese**									
Feta	13.5	1.0	0.0	3.5	11.8	23.3	76.9	0.0	117
**Italian Cheeses**									
Mozzarella	15.0	53.1	1.6	0.0	0.0	0.0	7.5	0.0	62.2
Parmesan (grana padano)	20.6	0.0	0.0	0.5	1.0	1.5	0.0	0.0	3
Gorgonzola	17.3	111	0.0	1.7	30.7	2.4	2.5	5.1	153
Pecorino	55.6	93.7	0.0	0.0	0.0	0.0	0.0	0.0	93.7
**Swiss Cheeses**									
Emmenthal	24.1	89.5	21.5	0.0	0.0	0.0	0.0	322	433
Gruyère	25.0	51.5	13.8	0.0	0.0	0.0	0.0	0.0	65.3
Raclette	15.5	47.7	4.0	3.1	11.3	47.7	209	0.0	323
**Norwegian Cheeses**									
Gamalost	1.8	10.3	6.2	2.9	9.7	51.2	440	22	542
Norvegia	43.7	51.0	0.0	3.0	13.3	52.5	295	0	415

Each value is the average value of duplicate measurements with a variation coefficient < 15%.

**Table 3:** Vitamin K content of non-dairy foods.

Type of Food	Vitamin K_1_ (ng/g)	MK-4 (ng/g)	MK-5 (ng/g)	MK-6 (ng/g)	MK-7 (ng/g)	MK-8 (ng/g)	MK-9 (ng/g)	MK-10 (ng/g)	Total Vitamin K_2_ (ng/g)
**Meat**									
Minced meat	10.9	76.1	0.0	0.0	0.0	0.0	0.0	0.0	76.1
Pork cutlet	0.0	10.5	0.0	1.1	0.0	0.0	0.0	0.0	11.6
Beef (meat)	0.2	13.9	0.0	0.0	1.3	3.7	0.0	0.0	18.9
Beef (liver)	22.9	2.4	0.0	11.2	49.9	16	14.6	18.3	112
Pork (meat)	0.0	13.6	0.0	0.0	0.0	0.0	0.0	0.0	13.6
Pork (liver)	0.0	2.8	0.0	10.5	5.1	0.0	0.0	0.0	18.4
Chicken (meat)	0.0	101	0.0	0.0	0.0	0.0	0.0	0.0	101
Deer (back)	24.3	8.8	0.0	0.0	0.0	0.0	0.0	0.0	8.8
**Fish**									
Mackerel	5.1	6.2	0.0	0.0	0.0	0.0	0.0	0.0	6.2
Eel	13	631	0.0	0.0	0.0	0.0	0.0	0.0	631
Plaice	0.0	3.8	0.0	0.0	0.0	49	0.0	0.0	52.8
Prawns	0.0	1.9	0.0	0.0	0.0	0.0	0.0	0.0	1.9
Salmon	1.3	5.7	0.0	0.0	0.0	0.0	0.0	0.0	5.7
Herring	1.1	0.7	0.0	0.0	0.0	0.0	0.0	0.0	0.7
**Vegetables**									
Natto	321	0.0	72	124	9965	824	0.0	0.0	10985
Sauerkraut	224	4.3	8.6	15.9	2.3	8.9	15	0.0	55

Each value is the average value of duplicate measurements with a variation coefficient < 15%.
